# Unpacking Hispanic Ethnicity—Cancer Mortality Differentials Among Hispanic Subgroups in the United States, 2004–2014

**DOI:** 10.3389/fpubh.2018.00219

**Published:** 2018-08-31

**Authors:** Dinorah Martinez Tyson, Patricia Medina-Ramirez, Ann M. Flores, Rebecca Siegel, Claudia Aguado Loi

**Affiliations:** ^1^Department of Community and Family Health, University of South Florida, Tampa, FL, United States; ^2^Department of Health Outcomes & Behavior, Moffitt Cancer Center, Tampa, FL, United States; ^3^Feinberg school of Medicine, Northwestern University, Chicago, IL, United States; ^4^Surveillance Information Services, American Cancer Society, Atlanta, GA, United States; ^5^Department of Health Sciences and Human Performance, University of Tampa, Tampa, FL, United States

**Keywords:** cancer, Hispanic Americans, Latinos, health disparities, mortality, minority health

## Abstract

**Introduction:** National data on the epidemiology of cancer are commonly reported by broad racial/ethnic categories, such as “Hispanic.” However, few studies have disaggregated Hispanic groups and explored mortality differentials in this heterogeneous population. This paper aims to further examine cancer mortality differentials among Hispanic subgroups in the U.S.

**Materials and Methods:** The study examined cancer deaths in the United States from 2004 to 2014 among decedents classified as Mexican, Puerto Rican, Cuban, Dominican, Central/South American and non-Hispanic white on the death certificate among those who were 20 years or older at the time of death. Data were obtained from the National Vital Statistics System. Sex-specific age-adjusted mortality rates were computed for a 10-year period and each individual year, for all cancers combined. Differences by age group, cancer sites, and age distribution were also assessed.

**Results:** A total of 296,486 Hispanic cancer deaths were identified. Mortality rates of the Hispanic subgroups compare favorably with those of non-Hispanic whites. The mortality rates for Mexicans are very similar to those of all Hispanics combined, whereas the rates for Cuban and Puerto Ricans are higher. Dominicans and Central/South Americans had the overall lowest mortality rates. Statistically significant decreases in cancer mortality rates were noted in some sub-groups, but rates increased among Dominican women. Age-adjusted mortality rates by cancer site varied among Hispanics subgroups and gender. Among Cubans, only 5% of cancer deaths occurred before the age of 50 compared to 16% of cancer deaths among Central/South American.

**Conclusion:** While it is common to present data on the burden of cancer among Hispanics as an aggregate group, this study illustrates that the burden of cancer varies by Hispanic subgroups. The disaggregation of Hispanics by ancestry/country of origin allows for a clearer understanding of the health status of this growing population and is needed if health disparities are to be adequately identified, understood and addressed.

## Introduction

The Hispanic/Latino population in the United States, projected to make up 31% of the population by 2060, is comprised of a diverse group of individuals who trace their heritage to more than 20 Spanish-speaking countries worldwide, regardless of race (1, 2). The term Hispanic is the ethnic classification used in federal and state reporting systems which was created by the Office of Budget and Management (OBM) and is usually assessed by selecting one of two categories: Hispanic or Latino and not Hispanic or Latino (3). The terms Latino and Hispanic are often used interchangeably as we have done in this paper.

Increase in the U.S. Latino population is propelled by both a greater number of U.S. born Latinos and ongoing immigration to the U.S. from Spanish-speaking countries (4, 5). When compared to the non-Hispanic white population, Hispanics tend to experience greater health disparities as a result of structural, socio-demographic, psychosocial, and cultural factors (6, 7). For example, approximately 24% of Hispanics live below the federal poverty line (8, 9) and 35% have less than a high school education (10). Additionally, in 2012, one-third of U.S. Latinos had no health insurance (11) and reported not having a consistent health care provider, with half of the population reporting no contact with a health care provider in the previous 6 months (12). These statistics vary depending on Hispanic country of origin, years living in the U.S., and nativity status (U.S. born vs. foreign born) (13). The rapid growth of the Latino population, combined with the documented health disparities experienced by this group and diversity within this Hispanic population, will have a critical impact on cancer incidence and mortality.

Although Hispanics have an overall lower incidence rate for all cancers combined (9), they are more likely to be diagnosed with advanced stages of the disease and experience lower survival as compared to non-Hispanic whites (11, 14, 15). Moreover, they are at higher risk of infection-related cancers such as stomach and liver (16). Determinants of health that contribute to this disparity include low rate of medical insurance coverage (17, 18), lack of knowledge about services (19), scarce availability and access to genetic counseling (20), limited access to and use of cancer screening programs (9, 21, 22), poverty, neighborhood socioeconomic status, (23, 24) and other complex sociocultural and geographic factors, such as living in an ethnic enclave (25–27). Distinct from non-Hispanic whites, Siegel et al. (28) reported cancer as the leading cause of death for Hispanics, surpassing heart disease (28). The leading causes of cancer death among Hispanic women are breast cancer (16%), followed by lung (13%) and colorectal (9%) cancers. Among Hispanic men, the leading cause of cancer death is lung cancer (17%), followed by colorectal (11%) and prostate (9%) cancers (29).

The available national data on the epidemiology of cancer among diverse racial/ethnic groups are commonly reported by broad racial/ethnic categories such as “Hispanic.” Few studies have investigated differences by Hispanic subgroup (16, 30). Existing studies have considered rates in specific states such as Florida (16, 31), but do not include all Hispanic subgroups (29, 32), or focus on only one specific cancer type (33).

Given the diversity of the populations that fall within this broad ethnic group, the U.S. Hispanic population serves as an illustrative example to explore the heterogeneity found within socially constructed categories. Thus, this paper examines cancer mortality differentials among Latino subgroups in the U.S. to help guide prevention and treatment efforts that will ultimately impact survivorship. Specifically, the aims of this study, which extend our previous state-level report to the national landscape, are to (1) examine cancer mortality rates among Latino/Hispanic subgroups and (2) compare mortality rates of Latino/Hispanic subgroups to non-Hispanic whites. To our knowledge it is the first study to examine cancer mortality among Hispanic subgroups including Dominicans and Central/South Americans in addition to Mexicans, Cubans, and Puerto Ricans in the United States.

## Materials and methods

The study examined cancer deaths in the United States (U.S.) from 2004–2014 among cases classified as Mexican, Puerto Rican, Cuban, Dominican, Central/South American and non-Hispanic white on the death certificate among those who were 20 years or older at the time of death. Death certificate data were obtained from the National Vital Statistics System's public-use data files supported by the National Center for Health Statistics (NCHS) (34). More than 99% of deaths occurring in the U.S. are registered in this system and this system is considered a fairly complete mortality data source (35). Cause of death was defined according to International Classification of Disease codes, 10th revision (ICD-10) (36). Decedent age, race, educational attainment, and marital status were also collected from the death certificates. Identification of Hispanic ethnicity was based on the information recorded on the death certificate following one of two formats recommended by the NCHS (37). Previous studies have shown that Hispanic ethnicity data from death certificates is accurately ascertained, in part because death certificate data is normally completed with the assistance of family members or friends of the deceased, increasing the accuracy of Hispanic classification (38, 39). This research was deemed exempt by the University of South Florida Institutional Review Board.

### Calculation of rates and analysis

Overall cancer mortality rates were computed for each Hispanic subgroup (Mexican, Puerto Rican, Cuban, Dominican, and Central/South American) and non-Hispanic whites for the overall study period (2004–2014), for each individual year, by sex, and using 5-year age group (from 20–25 to 80–85 and 85+ years). Single-year population estimates from the U.S. Census Bureau American Community Survey Public Use Microdata Sample (PUMS) were used as denominator data (40). Rates were standardized using the 2000 U.S. standard population. Joinpoint (41) analyses were applied to test the trends in annual percentage change for statistical significance. The log transformation was used to analyze the trends of age standardized rates. Age-adjusted, sex-specific rates were also calculated for the top 10 cancer types for all years combined (2004–2014). SAS statistical software (version 9.4; SAS Institute Inc, Cary, NC) and Microsoft Excel were used for the secondary data analyses.

## Results

During 2004–2014, a total of 296,486 cancer deaths occurred among decedents of Hispanic origin aged 20 or older, of which 60% (*n* = 178,926) were Mexican, 13.7% (*n* = 40,766) were Puerto Rican, 12.3% (*n* = 36,377) Central/South American, 11.1 % (*n* = 32,976) as Cuban, and 2.5% (*n* = 7,441) Dominican. During the same time period, 5,039,241 non-Hispanic whites died from cancer in the United States (see Table 1). The mean age at death for non-Hispanic white women and men was 72 (SD, 13.4) and 71 (SD, 12.5) years respectively. Within the Hispanic subgroups, mean age was lowest among Mexicans. Cubans, on the other hand, had a mean age closer to non-Hispanic whites. A large majority of the decedents in the Hispanic subgroups were racially categorized as white (ranging from 91.3 to 99.1%). A larger proportion of Mexican and Dominican decedents (80.9% in both cases) were reported to have a high school education or less compared to 68.4% of Cubans, 78.3% Puerto Ricans, 66.5% of Central/South Americans and 59.4% of non-Hispanic whites.

**Table 1:** Decedent demographics by Hispanic origin adults 20 years and older 2004–2014.

	**Mexican**		**Puerto Rican**		**Cuban**		**Dominican**		**Central/South American**		**Hispanic**		**White non-Hispanic**	
	***n***	**%**	***n***	**%**	***n***	**%**	***n***	**%**	***n***	**%**	***n***	**%**	***n***	**%**
Number of decedents	178,926	60.3	40,766	13.7	32,976	11.1	7,441	2.5	36,377	12.3	296,486	5.6	5,039,241	94.4
Men	95,452	53.3	21,868	53.6	18,710	56.7	3,395	45.2	16,425	45.2	155,850	52.6	2,637,429	52.3
Women	83,474	46.7	18,898	46.4	14,266	43.3	4,046	54.8	19,952	54.8	140,636	47.4	2,401,812	47.7
**MEAN AGE (SD)**														
Men	66 (15.0)		67 (13.4)		72 (12.2)		67 (14.1)		65 (15.6)		68 (14.6)		71 (12.5)	
Women	66 (15.7)		67 (14.7)		74 (13.33)		67 (15.2)		66 (15.50)		67 (15.5)		72 (13.4)	
**MARITAL STATUS**														
Married	98,986	55.3	17,640	43.3	16,194	49.1	3,576	48.1	19,289	53.0	155,685	52.2	2,602,285	51.6
Other	79,940	44.7	23,126	56.7	16,782	50.9	3,865	51.9	17,088	47.0	140,801	47.8	2,436,956	48.4
**RACE**														
White	177,231	99.1	39,667	97.3	32,134	97.5	6,793	91.3	34,695	95.4	290,520	97.6	5,039,241	100
Black	565	0.3	712	1.8	782	2.4	634	8.5	1,471	4.0	4,164	1.5	0	0
Other	1,130	0.6	387	1.0	60	0.2	14	0.2	211	0.6	1,802	1.0	0	0
Educational attainment^*^	154,071		33,796		29,576		7,441		30,618		247,615		3,169,375	
Less than HS	86,740	56.3	12,939	38.3	10,230	34.6	3,083	41.4	10,213	33.4	123,205	46.8	518,233	16.4
High School	37,941	24.6	13,501	40.0	10,001	33.8	2,936	39.5	10,126	33.1	74,505	29.7	1,363,748	43.0
Some College	18,690	12.1	3,915	11.6	3,410	11.5	562	7.6	4,785	15.6	23,475	12.8	621,965	19.6
College graduate	5,218	3.4	1,712	5.1	3,230	10.9	459	6.2	3,178	10.4	13,797	5.6	374,530	11.8
Graduate degree	2,494	1.6	873	2.6	1,861	6.3	188	2.5	1,639	5.4	7,055	2.9	218,151	6.9
Unknown	2,988	1.9	856	2.5	844	2.9	213	2.9	677	2.2	5,578	1.2	72,748	2.3

* *There was some missing data for education thus the n is smaller than the total sample*.

*Table excludes cases classified as other/unknown Hispanics and Spaniards*.

*Source: National Center for Health Statistics (Compressed Mortality File 2004–2014)*.

### Mortality differentials among hispanics

As an aggregate group, both male and female Hispanics' overall, cancer mortality rates (208.7, 95% CI 205.6–211.8 Male; 140.2, 95% CI 137.8–142.5 Female), are statistically significantly different from non-Hispanic whites (303.1, 95% CI 301.9–304.2 Males; 214.1, 95% CI 213.1–215.0 Females). The mortality rates of the Hispanic subgroups are also significantly different when we compare them to each other (see Table 2).

**Table 2:** Age-adjusted cancer deaths rates for all cancer types Combined, per 100,000, 2004-2014 (11 years).

	**Rate**	**95% CI**		**SE**
		**L**	**U**	
**MEN**				
Mexican	205.4	201.3	209.5	2.1
Puerto Rican	237.2	227.0	247.4	5.2
Cuban	234.8	224.0	245.5	5.5
Dominican	144.8	129.4	160.2	7.9
Central/South American	162.4	154.3	170.4	4.1
Hispanic	208.7	205.6	211.8	1.6
White non-Hispanic	303.1	301.9	304.2	0.6
**WOMEN**				
Mexican	142.0	138.8	145.1	1.6
Puerto Rican	151.5	144.4	158.6	3.6
Cuban	139.5	131.5	147.6	4.1
Dominican	99.3	89.2	109.3	5.1
Central/South American	119.1	113.6	124.5	2.8
Hispanic	140.2	137.8	142.5	1.2
White non-Hispanic	214.1	213.1	215.0	0.5

*Source: National Center for Health Statistics (Compressed Mortality File 2004–2014)*.

**Figure 1:**
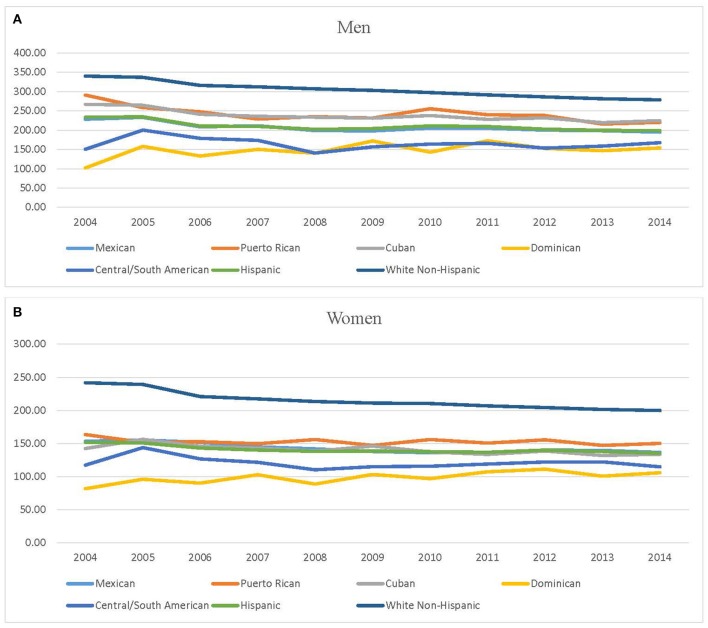
**(A,B)** Cancer mortality trends 2004−2014. **(A)** Cancer mortality trends among men decedents. **(B)** Cancer mortality trends among women decedents. Source: National Center for Health Statistics (Compressed Mortality File 2004–2014).

Cancer mortality trends from 2004-2014 are presented in Figures 1A,B. Table 3 shows the results of Joinpoint trend analysis by Hispanic subgroups. During the study years, statistically significant decreasing trends in cancer mortality rates were observed for male in all the Hispanic subgroups with the exception of Dominican and Central/South American. For Mexican and Puerto Rican age-adjusted rates there was a significant downward trend of −1.3% and −1.7%, respectively. For Cuban there was one Joinpoint: the rate decreased by 4.2% from 2004 to 2007 and by 0.8% (non-significant) from 2007 to 2014. (see Figures 1A,B). For female, statistically significant decreasing trends were also observed for all Hispanic subgroups excluding Puerto Rican and Central/South American. There was one Joinpoint for Mexican at 2010: the rate decreased by 2.2% per year from 2004 to 2010 and increased by 0.4 % (non-significant) from 2010 to 2014. For Dominicans between 2004 and 2014 there was a significant upward trend of 1.9%. For Cubans rates decreased 1.2% per year between 2004 and 2014.

**Table 3:** Joinpoint regression analysis by Hispanic subgroup and sex 2004–2014.

	**Trend 1**			**Trend 2**					
	**Year**	**APC**	**95% Confidence Interval**	**Year**	**APC**	**95% Confidence Interval**	**Year**	**AAPC**	**95% Confidence Interval**
**MALE**									
Mexican	2004–2014	−1.3^*^	(−2.1, −0.6)				2004–2014	−1.3^*^	(−2.1, −0.6)
Puerto Rican	2004–2014	−1.7^*^	(−2.9, −0.5)				2004–2014	−1.7^*^	(−2.9, −0.5)
Cuban	2004–2007	−4.2^*^	(−8.2, −0.1)	2007–2014	−0.8	(−1.8, 0.3)	2004–2014	−1.8^*^	(−3.0, −0.7)
Dominican	2004–2014	1.7	(−0.8, 4.3)				2004–2014	1.7	(−0.8, 4.3)
Central/South American	2004–2014	−0.7	(−2.7, 1.3)				2004–2014	−0.7	(−2.7, 1.3)
Hispanic	2004–2007	−4.2	(−9.1, 0.9)	2007–2014	−0.5	(−1.7, 0.7)	2004–2014	−1.6^*^	(−3.0, −0.2)
White Non-Hispanic	2004–2006	−3.7^*^	(−4.6, −2.8)	2006–2014	−1.7^*^	(−1.8, −1.6)	2004–2014	−2.1^*^	(−2.3, −1.9)
**FEMALE**									
Mexican	2004–2010	−2.2^*^	(−3.2, −1.2)	2010–2014	0.4	(−1.2, 2.1)	2004–2014	−1.2^*^	(−1.9, −0.5)
Puerto Rican	2004–2014	−0.4	(−1.0, 0.2)				2004–2014	−0.4	(−1.0, 0.2)
Cuban	2004–2014	−1.2^*^	(−1.9, −0.5)				2004–2014	−1.2^*^	(−1.9, −0.5)
Dominican	2004–2014	1.9^*^	(0.5, 3.4)				2004–2014	1.9^*^	(0.5, 3.4)
Central/South American	2004–2014	−0.8	(−2.2, 0.6)				2004–2014	−0.8	(−2.2, 0.6)
Hispanic	2004–2007	−3.1^*^	(−5.4, −0.6)	2007–2014	−0.3	(−0.9, 0.3)	2004–2014	−1.1^*^	(−1.8, −0.5)
White Non-Hispanic	2004–2007	−3.9^*^	(−5.8, −1.9)	2007–2014	−1.1^*^	(−1.7, −0.6)	2004–2014	−2.0^*^	(−2.5, −1.4)

* *APC and AAPC are significantly different from zero (P < 0.05). APC, Average Percent Change. AAPC, Annual Average Percent Change*.

### Mortality rates by cancer site for hispanic subgroups

The top 10 causes of cancer death for each Hispanic subgroup and Hispanics overall are listed in Appendix A. Among men, seven out of the top 10 cancers were found across all the Hispanic subgroups (lung/bronchus, prostate, colorectal, pancreas, leukemia, lymphoma, and liver). When examining all deaths attributable to cancer among women, nine out of the top 10 cancers were found across all Hispanic subgroups (breast, lung/bronchus, colorectal, pancreas, ovarian, leukemia, lymphoma, uterine, and liver). Please see Appendix A.

Age adjusted mortality rates by cancer site per 100,000 varied among Hispanics subgroups in this study. The rate for lung cancer deaths for Cuban males was 69.96 compared to 28.75 among Central/South American decedents. However, the highest mortality rates were for non-Hispanic white men (93.49). Liver cancer mortality rates were highest for Puerto Rican men at 24.03 and lowest for Dominicans at 10.41. Colorectal cancer mortality rates were highest for non-Hispanic white men. Across Hispanic subgroups, the rate for colorectal cancer was highest for Puerto Rican men at 25.68, followed by Cuban men at 25.08 and were lowest among Dominican men at 13.25. Head and neck mortality rates were highest among Puerto Rican men at 5.46 per 100,000.

Among women, non-Hispanic white women had the highest cancer mortality rate per 100,000 population for lung cancer at 59.99 compared to 20.05 all Hispanics, 24.78 in Puerto Rican, 23.18 in Cuban, 19.10 in Mexican, 14.47 in Central South American, and 13.57 in Dominican female decedents. Similar to the male decedents Cuban and Puerto Rican women also had the higher rates of colorectal cancer deaths for Hispanic subgroups at 17.25 and 17.27, respectively. In contrast, the colorectal cancer mortality rate for Dominican female decedents was 9.41. Cervical cancer rates were highest for Puerto Rican women at 4.78 compared to 2.56 among Cuban women decedents. However, the cervical cancer mortality rates among Mexican women were very similar to Puerto Rican women at 4.30. There was also considerable variation in breast cancer mortality rates across the Hispanic subgroups. It was highest for Cuban and Puerto Rican females at 23.34 and 23.47 followed by Mexican at 20.62, Central/South American women at 16.03 and Dominicans at 15.60. However, the breast cancer mortality rate was highest for non-Hispanic white women at 31.70. Among the Hispanic subgroups, stomach cancer mortality rates were lowest among Cuban women (2.81) and highest among Central/South American women decedents (7.65).

### Age and cancer

**Figure 2:**
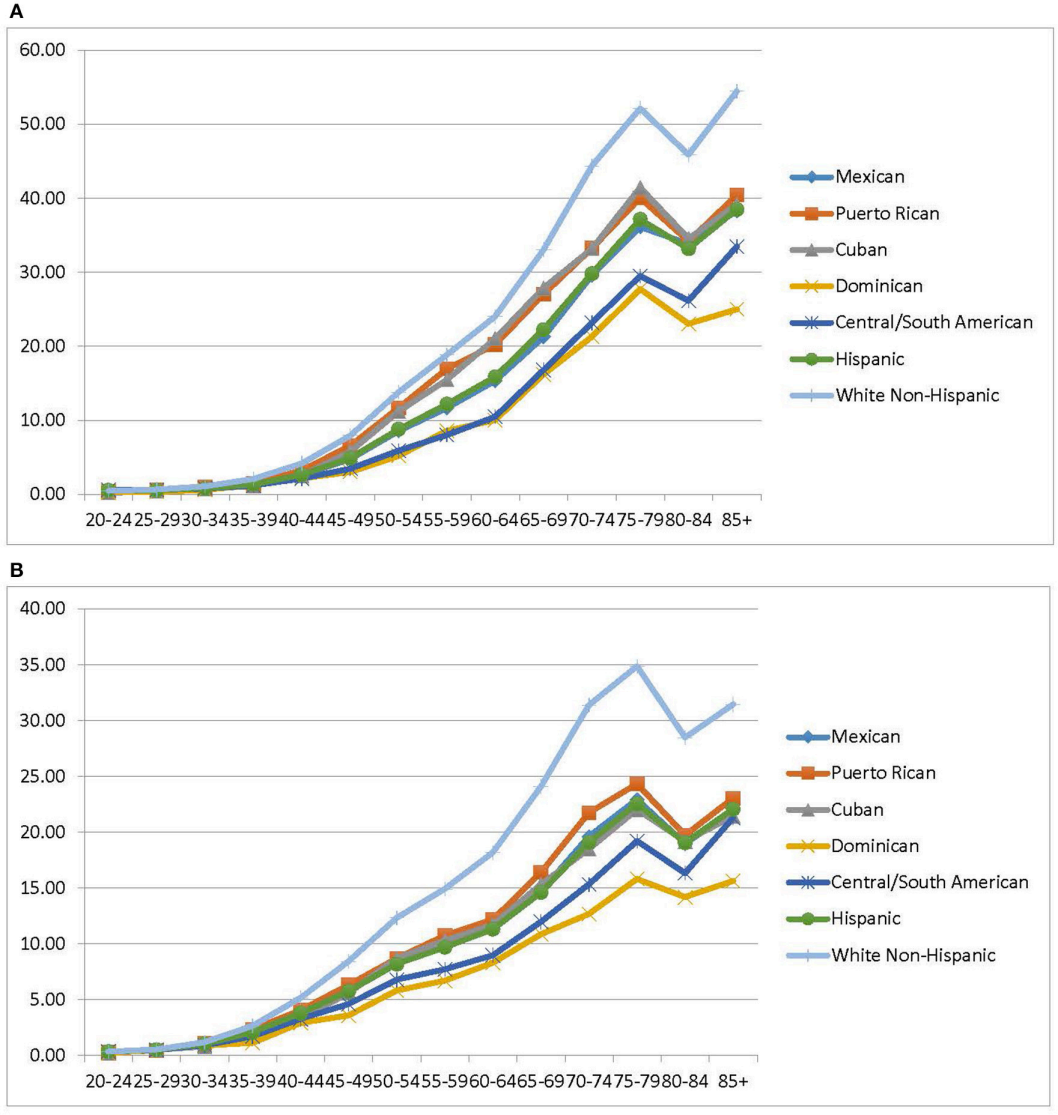
**(A)** Age-specific cancer mortality rates males, 2004–2014. **(B)** Age-Specific cancer mortality rates females, 2004–2014. Source: National Center for Health Statistics (Compressed Mortality File 2004–2014).

Cancer mortality risk increases with age. When Hispanics are disaggregated, age-specific risk patterns vary by subgroup. Figures 2A,B show the age-specific, cancer mortality rates by 5-year age group for men and women. Compared to the Hispanic subgroups, the trend among non-Hispanic whites is considerably higher. Cubans and Puerto Ricans have a similar pattern of cancer death risk by age, which is higher than that of Hispanics and Mexicans, in contrast to Central/South American and Dominican.

**Figure 3:**
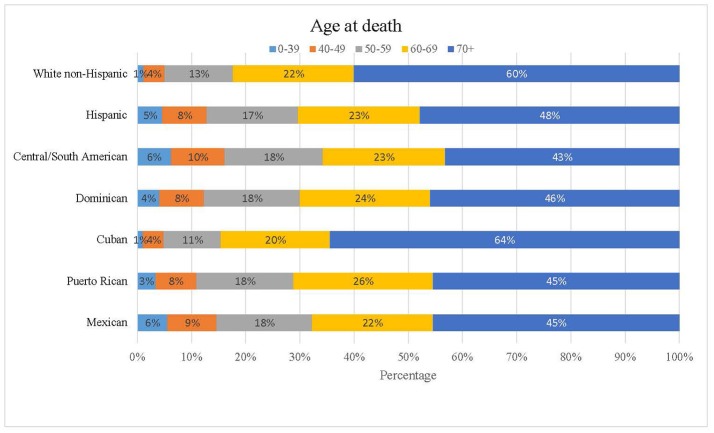
Proportion of cancer deaths by age. Source: National Center for Health Statistics (Compressed Mortality File 2004–2014).

The proportion of cancer deaths by age are presented in Figure 3. Cubans and non-Hispanic whites have a similar age distribution of cancer mortality, which approximately 60% of deaths occurring over the age of 70. In contrast, about one-third of deaths among Central/South American, and Mexican occurred under the age of 59.

## Discussion

By computing cancer mortality rates for Hispanic subgroups, rather than for all Hispanics combined, we are able to assess and compare differences that would otherwise be masked. As expected, the age-adjusted mortality rates for all Hispanics combined were lower as compared to non-Hispanic whites. Similarly, the mortality rates of the Hispanic subgroups compare favorably with those of non-Hispanic whites. The mortality rates for Mexicans are very similar to those of all Hispanics combined, whereas the rates for Cuban and Puerto Ricans are higher. Dominicans and Central/South Americans had the overall lowest mortality rates among all the Hispanic subgroups. For each selected Hispanic subgroup, the rates were lower for women than in men. The factors contributing to these differences are complex and multifaceted, often rooted in a number of biological, socio-cultural, structural, and behavioral explanations.

Previous studies have shown greater incidence rates of infection-related cancers among Hispanics, specifically stomach cancers in Mexican and Central/South Americans (42). In our study, mortality rates for infection-related cancers, such as stomach cancer were particularly high among Mexican and Central/South Americans as well. As noted in other research infection of *Helicobacter pylori (H. pylori)*, specifically a pattern of seroprevalence of *H. pylori*, is high among this subgroup of Hispanics and may be an important explanation for our findings (43). Patterns in nutritional patterns and genetic factors are other possible explanations for higher stomach cancer mortality rates. Specifically, dietary salt intake, increased rates of obesity, and certain genetic risk factors vary across racial group (44).

Other infections-related cancers from hepatitis B, C, HPV and HIV (e.g., liver, oropharyngeal, cervical, anal, vaginal, vulvar, penile Kaposi' sarcoma, non-Hodgkin, and Hodgkin's lymphoma) were not observed with the exception of higher cervical cancer mortality rates among Mexican and Puerto Rican women subgroups and liver cancer among Hispanics as a group (16, 29, 30). The Annual Report to the Nation on the Status of Cancer, 1975–2012, featured the increasing incidence of infection-related and liver cancers (45). These cancer types are associated with higher levels of alcohol consumption as well as obesity and diabetes (45, 46). In addition, viral hepatitis (HBV and HCV) are known causes of hepatocellular cancer for Hispanics born in the US and among the foreign born (45, 47).

The effect of acculturation on Hispanic health may help explain some of the observed differences. The concept of acculturation varies in degree and how it is operationalized, but it usually refers to the degree one adapts to a different culture and set of values, beliefs, and practices (48). Studies have shown that cancer-related mortality rates for Hispanics born in the US are comparable to those of non-Hispanic whites, while that is not the case for foreign-born individuals (30). There is evidence that greater acculturation has a negative influence on smoking, alcohol consumption, nutrition and diet, all of which are associated with poor health outcomes. On the other hand, greater acculturation seems to have a positive influence on access to health care and the use of preventive health services (49). For example, 76.9% of Mexicans who have been in the U.S. less than 10 years do not have health insurance compared to 47% of Mexicans who have been in the US for more than 10 years (28). Different degrees of acculturation can be related to different health outcomes depending on type of cancer: Hispanic Caribbean's, among which Dominicans are categorized, who migrated to Florida have higher mortality rates for stomach and liver cancer compared to US-born counterparts, while they have lower rates for breast, lung and pancreas cancers (50). Additionally, cancer-related beliefs and knowledge that are specific to each subgroups and socio-cultural context may affect access to services (15, 22, 51–53).

Health behaviors also vary by Hispanic subgroup (22). For example, Mexicans and Central Americans are among the Latino subgroups with the lowest rates of current smokers (54, 55). Historically, smoking is highest among Cubans (55) but more recent surveys suggest that this has changed. For example, studies conducted during 2010–2013 by the National Survey on Drug Use and Health reflects a higher percentage of Puerto Ricans (28.5%) who reported smoking in the last 30 days compared to 19.1% of Mexicans and 15.6% of Central/South Americans respectively (56). More recently, the smoking rate among Puerto Ricans (37.1) again surpassed that for Cubans (33.9%) (57). The higher mortality rates currently in Cubans reflect historically higher smoking prevalence; however, this trend may change in the future. Prevention efforts must be responsive to the heterogeneity of the Hispanic population while accounting for shared characteristics (e.g., language, cultural values) (58, 59).

Dietary behaviors vary among Hispanics by subgroup and geographic region as well (60). Being overweight or obese are highest among Puerto Ricans and Mexicans (54, 61). In addition, when compared to other Hispanic groups, Puerto Ricans have higher intake of fat and cholesterol (62, 63). Compared to other Hispanic groups, the lowest fruit and vegetable consumption was among Puerto Ricans in New York (64, 65). In a recent study by Penedo et al., when compared to Mexicans, Dominicans report better quality diet, which was associated with lower odds of cancer diagnosis (59). Physical inactivity levels have been noted to be highest among Cubans (54). When examined by tumor type, mortality rates for behavioral-related cancers, such as liver cancer were higher among Puerto Rican and Mexican men and women decedents, respectively.

Another factor that may contribute to the study findings could be rooted in notable differences in comorbidity, functional status and self-reported health quality. To illustrate, among Hispanic sub-groups, Puerto Ricans are most likely to report having co-occurring health conditions and are more likely to have functional impairments and least likely to report overall “good” or “excellent” health (54). In contrast, Mexicans report the overall best health status among Latino groups (54).

In general, Latinos are less likely than their non-Latino peers to report accessing preventive health services, such as screenings (29). Based on the 2011 American Community Survey English proficiency by Hispanic origin varies drastically. For example, 82% of Puerto Ricans are considered English proficient, compared to 66% of Mexicans and 60% of Cubans (2). Spanish-speaking Hispanics have increased barriers to accessing screening programs compared to English-speaking Hispanics (66). Differences in cancer screening rates across the Hispanic groups may partially explain the observed mortality differences (67). Mexican and Central American women have lower cancer screening rates (68, 69) which may contribute to higher proportion of deaths due to preventable and treatable cancers if detected early. In contrast, over 80% of Puerto Rican women report having had a Papanicolaou test, compared to 71.6% of Mexican women (70, 71), yet both had the highest cervical cancer mortality rates in our study. While Cuban women have the lowest rates of cervical cancer screening (less than 75%) (72) they also have lower cervical cancer mortality rates as the data show. Could some of these differences be attributed to higher rates of Human papillomavirus (HPV) among certain Hispanic groups? Research is needed to better understand these differences.

## Strengths and limitations

This research offers a significant contribution to the current literature on cancer among Hispanic population in the U.S. Comparative analyses were completed to highlight potential differences within the largest Hispanic subgroups in the U.S. and between non-Hispanic whites. Further, few studies report cancer outcomes for Dominicans nationwide, despite this being a growing segment of the Hispanic population for which cancer is an increasingly important health concern (15). Highlighting differences even in smaller subgroups, like Dominicans, is critical to reveal potential health disparities and to promote health equity for all.

However, several limitations to this paper must be acknowledged. Public use mortality data files from the National Center for Health Statistics were used for this study. This limited the availability of certain characteristics of interest that would enable regional and other demographic comparisons. For example, public use data files also did not have information about nativity (e.g., US born vs. foreign born). Last, the Central/South American Hispanic subgroup represents a large group with several Hispanic nationalities represented. Data from these subgroups were not systematically categorized of over the 10-year study period, and thus they were combined. Future research could explore differences further.

We must also consider the potential misclassification of Hispanic ethnicity (73, 74). Race and ethnicity are rarely uniformly classified on death certificates and medical records, especially when reporting ethnic and racial groups other than non-Hispanic whites and blacks (75, 76). Reporting of the race/ethnicity of the deceased on death certificates is done by the funeral director or physician based on either information gathered from a family member or friend (37). However, in the absence of family or friends, classification is based on observation (37). Despite the potential for misclassification of Hispanics, several studies have found high accuracy of birthplace data obtained from death certificates (77). Also, causes of death for minorities are more likely to be misclassified as “symptoms, signs, and ill-defined conditions” than they are for non-Hispanic whites (78, 79). The database we used for this study include all deaths registered in the U.S. The mortality rates for Hispanics are also affected by under coverage of the population during the census (80–82). Accuracy in the cause of death in cancer patients may also contribute to misclassification bias (77) which is difficult to discern in the database used for this study.

In summary, while it is common to present data on the burden of cancer among Hispanics as an aggregate group, this study illustrates that important differences in health indicators often used to measure health disparities, like mortality, may be masked. The disaggregation of Hispanics by ancestry/country of origin allows for a clearer understanding of the health status of this growing population and is needed if health disparities are to be adequately identified, understood and addressed. Explanations for the observed differences are multifaceted and complex. Future studies should further examine the behavioral and sociocultural context that drive the observed differences.

## Author contributions

DM conceived the idea, directed the project, and supervised the work; PM-R retrieved and analyzed the data; CA and RS, assisted with the analysis and the interpretation of the data; DM, CA, and AF, participated in drafting the manuscript and literature review that informed the introduction and discussion. All authors discussed the results and commented on the manuscript.

### Conflict of interest statement

The authors declare that the research was conducted in the absence of any commercial or financial relationships that could be construed as a potential conflict of interest.

## Appendix

**Appendix A:** Age adjusted mortality rates by top 10 cancer types by subgroup and 95% CIs, 2004–2014.

	**Mexican**		**Puerto Rican**		**Cuban**		**Dominican**		**Central/South American**		**Hispanic**		**White non-hispanic**	
	**Rate**	**95% CI**	**Rate**	**95% CI**	**Rate**	**95% CI**	**Rate**	**95% CI**	**Rate**	**95% CI**	**Rate**	**95% CI**	**Rate**	**95% CI**
**Men**														
Lung/Bronchus	41.89	(40.09–43.69)	53.13	(48.47–57.79)	69.96	(64.35–75.57)	31.05	(24.21–37.89)	28.75	(25.46–32.04)	44.94	(43.55–46.33)	93.49	(92.86–94.12)
Prostate	24.27	(23–25.54)	27.70	(24.6–30.8)	27.36	(24.38–30.34)	25.90	(20–31.8)	23.90	(21.06–26.74)	25.44	(24.48–26.4)	29.35	(29.06–29.64)
Colorectal	21.19	(19.88–22.5)	25.68	(22.37–28.99)	25.08	(21.63–28.53)	13.25	(8.59–17.91)	14.34	(11.95–16.73)	21.79	(20.79–22.79)	26.47	(26.12–26.82)
Liver	20.34	(19.09–21.59)	24.03	(20.97–27.09)	10.76	(8.6–12.92)	10.41	(6.35–14.47)	11.30	(9.22–13.38)	18.47	(17.57–19.37)	11.25	(11.01–11.49)
Pancreas	13.26	(12.24–14.28)	13.33	(10.94–15.72)	15.33	(12.64–18.02)	10.41	(6.33–14.49)	11.47	(9.37–13.57)	13.49	(12.73–14.25)	18.03	(17.76–18.3)
Stomach	10.64	(9.7–11.58)	11.07	(8.87–13.27)	6.18	(4.4–7.96)	6.19	(3.05–9.33)	13.15	(10.86–15.44)	10.45	(9.74–11.16)	5.38	(5.22–5.54)
Leukemia	9.47	(8.57–10.37)	10.82	(8.57–13.07)	11.23	(8.84–13.62)	8.33	(4.55–12.11)	8.96	(7.02–10.9)	9.93	(9.24–10.62)	16.65	(16.38–16.92)
Lymphoma	9.21	(8.33–10.09)	9.64	(7.54–11.74)	8.72	(6.6–10.84)	6.52	(3.19–9.85)	8.55	(6.69–10.41)	9.00	(8.35–9.65)	11.97	(11.73–12.21)
Kidney	8.47	(7.65–9.29)	4.70	(3.27–6.13)	6.37	(4.65–8.09)	1.63	(0.08–3.18)	4.36	(3.07–5.65)	7.15	(6.58–7.72)	8.37	(8.17–8.57)
Bladder	4.56	(3.97–5.15)	6.82	(5.19–8.45)	9.30	(7.44–11.16)	3.41	(1.21–5.61)	4.41	(3.16–5.66)	5.61	(5.14–6.08)	11.92	(11.7–12.14)
Myeloma	4.71	(4.1–5.32)	5.37	(3.88–6.86)	4.31	(3–5.62)	4.76	(2.13–7.39)	4.79	(3.46–6.12)	4.81	(4.36–5.26)	5.75	(5.59–5.91)
Head and neck	3.24	(2.73–3.75)	5.46	(3.95–6.97)	4.01	(2.56–5.46)	2.40	(0.56–4.24)	1.78	(0.96–2.6)	3.40	(3.01–3.79)	5.41	(5.25–5.57)
**Women**														
Breast	20.62	(19.42–21.82)	23.47	(20.65–26.29)	23.34	(19.97–26.71)	15.60	(11.64–19.56)	16.03	(14.05–18.01)	20.84	(19.96–21.72)	31.70	(31.33–32.07)
Lung/Bronchus	19.10	(18–20.2)	24.78	(22.06–27.5)	23.18	(20.08–26.28)	13.57	(9.98–17.16)	14.47	(12.65–16.29)	20.05	(19.23–20.87)	59.99	(59.54–60.44)
Colorectal	12.59	(11.67–13.51)	17.27	(14.92–19.62)	17.25	(14.58–19.92)	9.41	(6.37–12.45)	10.37	(8.78–11.96)	13.68	(12.97–14.39)	18.79	(18.52–19.06)
Pancreas	11.13	(10.29–11.97)	10.43	(8.65–12.21)	10.50	(8.56–12.44)	7.29	(4.74–9.84)	10.05	(8.56–11.54)	10.87	(10.28–11.46)	13.44	(13.22–13.66)
Liver	9.90	(9.1–10.7)	7.45	(5.94–8.96)	5.40	(3.97–6.83)	6.61	(4.16–9.06)	7.03	(5.76–8.3)	8.46	(7.93–8.99)	4.90	(4.76–5.04)
Ovarian	8.29	(7.53–9.05)	7.62	(6.05–9.19)	8.06	(6.14–9.98)	4.77	(2.63–6.91)	7.16	(5.85–8.47)	8.02	(7.47–8.57)	11.93	(11.71–12.15)
Leukemia	5.94	(5.27–6.61)	6.43	(4.94–7.92)	6.71	(4.91–8.51)	5.28	(2.91–7.65)	5.71	(4.49–6.93)	6.11	(5.62–6.6)	9.08	(8.88–9.28)
Stomach	6.43	(5.74–7.12)	5.13	(3.84–6.42)	2.81	(1.65–3.97)	3.25	(1.45–5.05)	7.65	(6.26–9.04)	6.00	(5.53–6.47)	2.71	(2.61–2.81)
Lymphoma	6.16	(5.51–6.81)	5.78	(4.39–7.17)	5.82	(4.21–7.43)	3.90	(1.88–5.92)	5.91	(4.73–7.09)	5.90	(5.45–6.35)	7.39	(7.23–7.55)
Uterine	4.72	(4.15–5.29)	5.91	(4.54–7.28)	5.42	(3.85–6.99)	4.39	(2.33–6.45)	4.39	(3.37–5.41)	4.87	(4.46–5.28)	5.81	(5.65–5.97)
Cervical	4.30	(3.75–4.85)	4.78	(3.49–6.07)	2.56	(1.33–3.79)	2.88	(1.16–4.6)	3.45	(2.53–4.37)	3.95	(3.56–4.34)	2.93	(2.81–3.05)
Myeloma	3.29	(2.84–3.74)	4.09	(3.01–5.17)	2.94	(1.88–4)	3.60	(1.91–5.29)	2.72	(1.94–3.5)	3.30	(2.97–3.63)	3.52	(3.42–3.62)
CNS	3.19	(2.7–3.68)	2.84	(1.84–3.84)	4.08	(2.63–5.53)	2.95	(1.21–4.69)	2.93	(2.07–3.79)	3.23	(2.88–3.58)	5.48	(5.32–5.64)

*Source: National Center for Health Statistics (Compressed mortality file 2004–2014)*.
